# Primary cardiac hydatid cyst presenting with massive pericardial effusion: a case report

**DOI:** 10.1186/s43044-020-00085-x

**Published:** 2020-08-17

**Authors:** Badre El Boussaadani, Hind Regragui, Hanae Bouhdadi, Hicham Wazaren, Intissar Ajhoun, Mohamed Laaroussi, Mohammed Cherti

**Affiliations:** 1 Cardiology B Department, Ibn Sina University Hospital Center, Rabat, Morocco; 2 Mohammed V University, Rabat, Morocco; 3 Cardiovascular Surgery A Department, Ibn Sina University Hospital Center, Rabat, Morocco; 4 Laboratory of Parasitology and Mycology, Ibn Sina University Hospital Center, Rabat, Morocco

**Keywords:** Pericardial hydatid cyst, Cardiac hydatidosis, *Echinococcus granulosus*, Pericardial effusion, Pericystectomy, Case report

## Abstract

**Background:**

Cardiac hydatidosis is a rare manifestation of *Echinococcus* infection. It represents 0.5 to 2% of hydatic disease (Mustafa et al., Can J Cardiol 22:2, 2006). The most common localization is the myocardium of the left ventricle but can also touch the right ventricle, atrium, pericardium, interventricular septum, and pulmonary artery. Clinical presentation is varied ranging from clinical latency or minor symptoms to cardiogenic shock and sudden death. The present case describes a primary pericardial hydatid cyst, a very exceptional localization of cardiac hydatidosis, which can lead to a delayed diagnosis or to an erroneous treatment that can expose the life of the patient to complications and death if it is not considered. Diagnosis can be established by cardiac imaging and hydatid serology. Therapy management should combine both surgery and medical treatment by albendazole or mebendazole.

**Case presentation:**

We report a 70-year-old woman from Sale, who was admitted for dyspnea New York Heart Association (NYHA) class IV evolving in a febrile context with signs of right heart failure related to a rupture of a primary pericardial hydatid cyst with pre-tamponade. The diagnosis was confirmed by echocardiography, computed tomography scan (CT scan), and hydatic serology, and the patient was operated and put on albendazole for 3 months with favorable clinical course.

**Conclusions:**

The aims of this article are to consider the diagnosis of cardiac hydatid cysts in the presence of pericardial effusion, especially if there is a prior history of hydatid disease, a contact with animals, or when it occurs in an endemic country, and to be able to make a differential diagnosis with cardiac imaging in order to avoid its complications and to guide the management.

## Background

Cardiac hydatidosis is a rare manifestation of *Echinococcus* infection. It represents 0.5 to 2% of hydatic disease [1]. It can touch different structures of the heart, but the most common localization is the myocardium of the left ventricle.

Herein, we report a 70-year-old woman who was admitted for dyspnea NYHA class IV evolving in a febrile context with signs of right heart failure related to a rupture of a primary pericardial hydatid cyst with pre-tamponade. The aim of this article is about the management of cardiac hydatidosis.

## Case presentation

A 70-year-old woman from Sale, with no history of previous hydatid disease, was admitted to our cardiology department for dyspnea NYHA class IV evolving in a febrile context for 2 days. Its hemodynamic profile showed a tachycardia at 120 bpm with a systemic blood pressure at the lower limit 95/55 mmHg. The clinical examination found the signs of right heart failure. Echocardiography revealed a pericardial effusion of great abundance in pre-tamponade with an intramural honeycomb pattern in the right ventricular wall.

The honeycomb pattern as a specific image usually found in hydatid disease prompted the investigation of hydatid serology which was explored by the ELISA technique with the ELISA DRG Diagnostic kit. The test came back positive up to 31.90 OD.

**Fig. 1:**
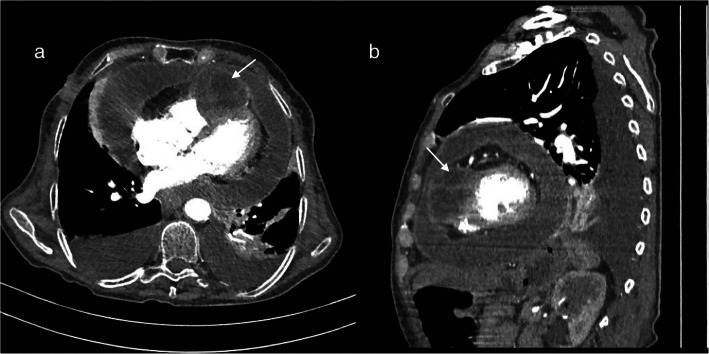
Appearance of massive pericardial effusion with hydatid cyst compressing the right ventricle (arrows) at CT scan. **a** Transversal view. **b** Sagittal view

The diagnosis of isolated cardiac hydatidosis involvement was established, and CT scan assessment showed a massive pericardial effusion with hydatid cyst compressing the right ventricle and mild bilateral pleural effusion (Fig. 1) with no other organ involvement.

**Fig. 2:**
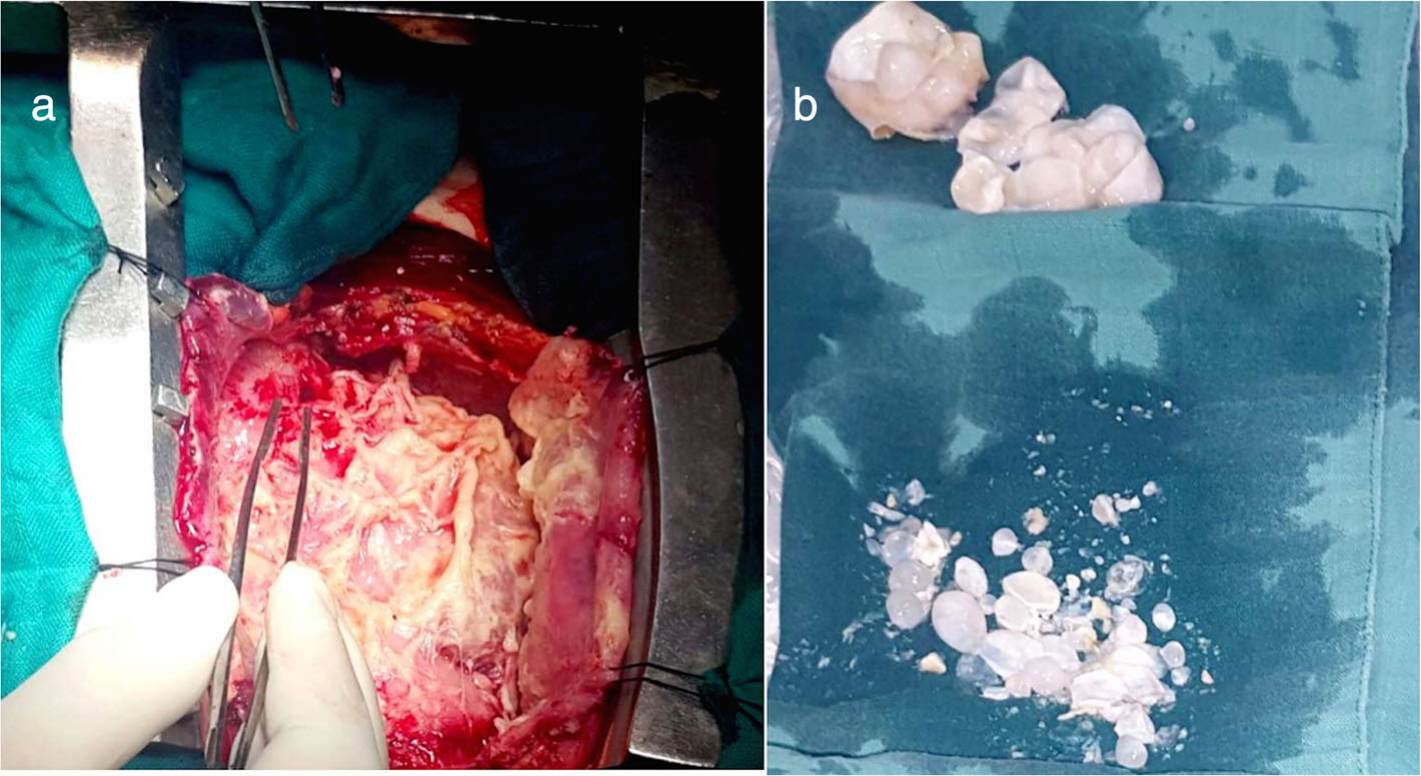
**a** Appearance of hydatid cysts of our patient during surgery. **b** Image of cyst formations after extraction

Surgery as indicated was realized through a median sternotomy with off-pump (Fig. 2). First, we proceeded to the aspiration of a pericardial liquid with whitish rock water appearance and to pericystectomy to remove the false membrane and cystic formations.

We proceeded after that to an off-pump treatment of the right ventricle cysts: we first used aspiration with a 10-G lumbar puncture needle connected directly to the aspirator, and then we removed the cystic membranes, the residual cavity was cleaned with gauze saturated with hypertonic sodium chloride and povidone-iodine solution, and was left open.

**Fig. 3:**
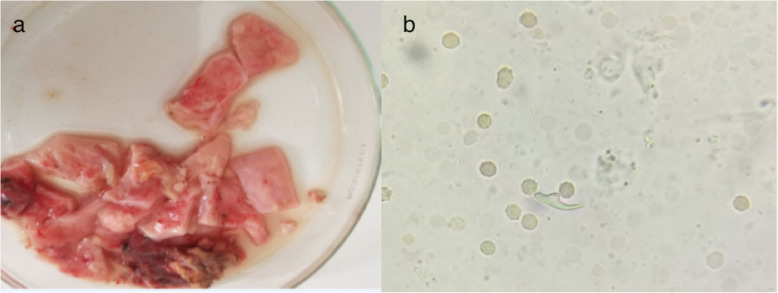
**a** Membrane of the emptied cyst. **b** *Echinococcus granulosus* hook under an optical microscope (× 400)

Part of the piece was sent to the parasitology laboratory. Examination under an optical microscope (× 400) had revealed rare hooks of *Echinococcus granulosus* (Fig. 3). The whitish rock water appearance of the pericardial liquid confirms the theory of intrapericardial cyst rupture. The patient was put on medical treatment with albendazole 10 mg/kg/day for 3 months with a favorable clinical course.

## Discussion

Hydatid disease is a larval cestodosis that remains endemic to Morocco. It is caused by a cestode of the *Echinococcus* family that mainly parasites dogs and other canids. It is usually located in the liver or lungs.

Cardiac hydatid cyst involvement is very uncommon and represents 0.5 to 2% of hydatic disease [1]. It is basically located at the left ventricle (60% of cases), right ventricle (10%), pericardium (7%), pulmonary artery (6%), left atrium (6%), and interventricular septum (4%) [2].

It is usually secondary to an extension of larvae through the systemic or pulmonary circulation or as a direct extension from near organs [3]. In our case, hydatid cyst disease was isolated to the pericardium and the right ventricle.

Our case report was about a 70-year-old patient, while the majority of cases in the literature were young adults [4]. She presented with fever and signs of right heart failure associated with pericardial effusion. In the other studies, the circumstances of discovery were varied depending on the size, location, number of the cyst, and the presence of complications, ranging from clinical latency to fever, chest pain, dyspnea, weakness, and at worst anaphylactic shock due to cyst rupture into the bloodstream or sudden death [1]. Other complications were described such as systemic or pulmonary hydatid embolism, valve obstruction, mitral regurgitation secondary to papillary muscle involvement, atrioventricular conduction defects, and arrhythmias [1]. The presentation of cardiac hydatidosis in our case and as described in the literature [1, 5, 6] was an intrapericardial rupture of the hydatid cyst with pre-tamponade.

Different methods can help for the diagnosis of cardiac hydatidosis, such as a prior history of hydatid disease, the presence of eosinophilia and abnormal shape of the heart shadow, or sometimes a calcified spherical mass at the chest X-ray [5]. Echocardiography, CT, and magnetic resonance imaging (MRI) findings can confirm the diagnosis. Echocardiography is the method of choice that shows the typical image of a uni- or multivesicular cyst, with a honeycomb appearance. The echolucent and multiseptate nature of hydatid cysts are charasteristic of the echinococcal cyst, but sometimes, it may be absent and should be considered in the differential diagnosis of cardiac tumoural lesions [5]. The hydatid serology was positive in our patient and often positive in the literature, but its negativity does not eliminate the diagnosis [7].

Surgery is of paramount importance because of the risk of hydatid cyst rupture. It consists of the excision of cysts and pericardial drainage if present. In addition, medical treatment with albendazole or mebendazole is recommended to reduce the risk of recurrence [3].

Postoperative, serological assessment is justified every 2 months for 2 years to detect a possible recurrence [7].

## Conclusion

The present case allows us to consider the diagnosis of cardiac hydatid cysts in the presence of pericardial effusion, especially if there is a prior history of hydatid disease or a contact with animals. Cardiac hydatidosis can be isolated or included with multiple organ disease. The problem lies in its complications which require both surgical and medical management.

## Data Availability

Data sharing is not applicable to this article as no datasets were generated or analyzed during the current study.

## Abbreviations

CT: Computed tomography
ELISA: Enzyme-linked immunosorbent assay
DRG: Diagnosis-related groups
MRI: Magnetic resonance imaging
NYHA: New York Heart Association
OD: Optical density
